# Intrafractional relationship changes between an external breathing signal and fiducial marker positions in pancreatic cancer patients

**DOI:** 10.1002/acm2.12841

**Published:** 2020-03-14

**Authors:** Niclas Pettersson, Oluwaseyi M. Oderinde, James Murphy, Daniel Simpson, Laura I. Cerviño

**Affiliations:** ^1^ Department of Radiation Oncology University of California San Diego La Jolla CA USA; ^2^ Department of Medical Physics and Biomedical Engineering Sahlgrenska University Hospital Göteborg Sweden; ^3^ Department of Radiation Physics Institute of Clinical Sciences The Sahlgrenska Academy University of Gothenburg Göteborg Sweden; ^4^ Department of Medical Physics Memorial Sloan Kettering Cancer Center New York NY USA

**Keywords:** fiducial markers, fluoroscopic imaging, pancreatic cancer, respiratory‐guided radiation therapy, SBRT

## Abstract

**Background and purpose:**

The purpose of this study of pancreatic cancer patients treated with respiratory‐guided stereotactic body radiotherapy (SBRT) on a standard linac was to investigate (a) the intrafractional relationship change (IRC) between a breathing signal and the tumor position, (b) the impact of IRC on the delivered dose, and (c) potential IRC predictors.

**Materials and methods:**

We retrospectively investigated 10 pancreatic cancer patients with 2–4 implanted fiducial markers in the tumor treated with SBRT. Fluoroscopic images were acquired before and after treatment delivery simultaneously with the abdominal breathing motion. We quantified the IRC as the change in fiducial location for a given breathing amplitude in the left–right (LR), anterior–posterior (AP), and superior–inferior (SI) directions from before to after treatment delivery. The treatment plans were re‐calculated after changing the isocenter coordinates according to the IRCs. Four treatment‐ or patient‐related factors were investigated as potential predictors for IRC using linear models.

**Results:**

The average (±1 SD) absolute IRCs in the LR, AP, and SI directions were 1.2 ± 1.2 mm, 0.7 ± 0.7 mm, and 1.1 ± 0.8 mm, respectively. The average 3D IRC was 2.0 ± 1.3 mm (range: 0.4–5.3 mm) for a median treatment delivery time of 8.5 min (range: 5.7–19.9 min; n = 31 fractions). The dose coverage of the internal target volume (ITV) decreased by more than 3% points in three of 31 fractions. In those cases, the 3D IRC had been larger than 4.3 mm. The 3D IRC was found to correlate with changes in the minimum breathing amplitude during treatment delivery.

**Conclusion:**

On average, 2 mm of treatment delivery accuracy was lost due to IRC. Periodical intrafractional imaging is needed to safely deliver respiratory‐guided SBRT.

## 1 INTRODUCTION

Respiration‐induced motion of pancreatic tumors can be substantial, and it affects all radiotherapy (RT) treatment steps from pretreatment imaging to dose delivery.^1^ While motion is present in all directions, it is usually largest in the superior–inferior (SI) direction, where the pancreas moves superiorly during the exhalation phase of the breathing cycle and inferiorly during the inhalation phase.^2^ The range of tumor motion in free‐breathing patients has been reported to be up to 6, 10, and 20 mm in the left–right (LR), anterior–posterior (AP) and SI directions, respectively.^3^ Creating and delivering treatment plans with dose distributions that cover the tumor would require large internal margins (IMs) to take the entire motion range into account. In stereotactic body radiotherapy (SBRT), this method would also result in irradiation of large volumes of the surrounding organ‐at‐risk (OARs) such as the stomach and the duodenum, to a high dose.^4^, ^5^ Therefore, to accurately treat the tumor while keeping the irradiation of the OARs within tolerance limits, a more complex motion management strategy is required.

One motion management strategy for pancreatic cancer SBRT is respiratory‐gated RT.^6^ By considering select phases of the breathing cycle (such as the end‐of‐exhale) during pretreatment imaging, treatment planning and delivery, there will be a decrease in total volume encompassed by the moving tumor. The patient’s breathing motion can be monitored by, for instance, measuring the external position of the abdominal or chest surface. This approach will decrease the volume of the required dose distribution to cover the tumor and, consequently, normal tissue irradiation will be reduced accordingly.^4^

During treatment setup, the tumor motion is observed (with, for instance, fluoroscopic imaging) simultaneously with the breathing motion, and a fraction‐specific relationship between the breathing signal and the tumor position is established to ensure that the planned dose distribution can be accurately delivered. The treatment delivery then is typically done using the breathing signal alone, and its accuracy is therefore dependent on the assumption that the relationship between the breathing signal and the tumor position that was established at setup also holds up during treatment delivery. This assumption may not hold, however, due to, for instance, muscle relaxation or movement of internal organs close to the tumor during treatment. The assumption has been extensively investigated for lung tumors,^7^, ^8^ but there are limited data available for pancreatic tumors. Malinowski et al. investigated pancreatic cancer patients treated with SBRT on a CyberKnife Synchrony.^9^ They found that the relationship between external markers and the fiducials degraded monotonically during treatment delivery and occurred in most pancreatic cancer patients after 30 min. Ge et al. reported that they detected decreased treatment accuracy in 15 of 40 investigated fractions and attributed changes to the external–internal relationship as the reason in at least 40% of cases for treatments delivered on a standard linac.^10^ They did not, however, report how large these changes were. Several aspects of how the treatment accuracy for respiratory‐gated RT holds up during treatment delivery remains to be investigated. One of the most important properties is how the shorter treatment delivery times typical for SBRT on standard linacs influence the consistency of the external–internal relationship. If larger margins between the internal target volume (ITV) and the planning target volume (PTV) are to be used to compensate for increased geometrical uncertainty, it is of interest to know whether the relationship changes are similar in all directions. Moreover, if one were aware of observable signs suggesting that the intrafractional relationship had substantially changed, treatment delivery could be halted, and the treatment setup redone before accurate treatment delivery started again.

In this study of patients with pancreatic cancer treated with SBRT on standard linacs, we investigated intrafractional relationship changes (IRC) between an external breathing signal and the positions of internal fiducial markers, and estimated its impact on the dose distribution in the ITV and OARs. We also investigated patient‐ and treatment‐related factors potentially predicting the occurrence of IRC.

## 2 METHODS AND MATERIALS

### 2.1 Patients and diagnostic imaging

We investigated 10 pancreatic cancer patients treated with SBRT at the University of California San Diego during 2016 and 2017, and who had two to four fiducials (diameter = 0.8 mm, length = 3 mm; MTNW887808, CIVCO Medical Solutions, Kalona, IA, USA) implanted in the tumor. The institutional review board approved the study. Patients underwent pretreatment free‐breathing CT scans (GE Lightspeed, GE Health Care, Pasadena, CA, USA) where the RPM system (Varian Medical Systems, Palo Alto, CA, USA) was used to monitor breathing motion externally. A phase‐based 4DCT was created by stratifying the breathing cycle into 10 phases in steps of 10%, where the 0% phase corresponds to the end of inhalation. The average (AVE) and the maximum intensity projection (MIP) CT image sets consisting of the 30% to 70% phases (CT_3070AVE_ and CT_3070MIP_) as well as all 10 individual phases were exported to the treatment planning system (TPS; Eclipse version 13.6, Varian Medical Systems, Palo Alto, CA, USA) for contouring and treatment planning.

### 2.2 Contouring and treatment planning

The ITV was contoured on the CT_3070AVE_ image series, and a PTV was created from the ITV using a 3‐mm isotropic margin. The CT_3070AVE_ images were also used for contouring of OARs. The OARs that were considered for the ten patients included in this study were the stomach, the duodenum, the spinal cord, the bilateral kidneys, and the liver. The fiducials were contoured on the CT_3070MIP_ images.

The planning objective for the PTV was to cover at least 95% of its volume with the prescription isodose (V_100%_ > 95%), whereas no objective was used for the ITV. The objective for both the stomach and the duodenum was that the dose received by 1 cm^3^ should be below the prescribed dose (D_1cm3_ < 100%). The spinal cord objective was D_1cm3_ < 8 Gy, the bilateral kidney objective D_75%_ < 12 Gy, and the liver objective D_700cm3_ < 15 Gy. In cases with conflicting objectives, trade‐offs could be made by the treating physician.

Volumetric‐modulated arc therapy (VMAT) treatment plans using two 180‐degree arcs, one with clockwise and one with anticlockwise rotation, were created using the treatment plan objectives above. The nominal photon beam energy was in most cases 6 MV using the flattening filter, but 6 and 10 MV flattening‐filter free beams were also used. All dose distribution calculations were performed in Eclipse with the analytical anisotropic algorithm (AAA; version 13.6.23) using a dose grid size resolution of 2.5 mm x 2.5 mm x 2.5 mm.

The SBRT was prescribed to be delivered in five fractions with a total dose ranging from 25.0 (5 × 5.0) to 45.0 (5 × 9.0) Gy. Digitally reconstructed radiographs (DRRs), including overlays of contoured fiducials, were calculated to assist in patient setup.

### 2.3 Treatment setup

The following procedure was utilized to treat each fraction using amplitude‐based respiratory‐gated RT on a TrueBeam linear accelerator (Varian Medical Systems, Palo Alto, CA, USA) including the RPM system. Setup and treatment delivery were done without breathing control and without visual coaching. The RPM box was placed on the patient, usually slightly below the sternum at the xiphoid process. The patient was then initially positioned by kilovoltage (kV) and cone‐beam CT (CBCT) imaging. For final positioning and setup of the gating window thresholds, simultaneous fluoroscopic imaging and breathing signal monitoring was performed. Observing the fiducials on the real‐time fluoroscopy and using the DRRs as reference, the gating window thresholds were set to allow the beam to be on only when the fiducials were positioned within the contours.

### 2.4 Data acquisition and analyses

When the patient setup was considered satisfactory, we made a simultaneous acquisition of the breathing motion signal and fluoroscopic image data in both AP and lateral (patient right‐to‐left) directions before treatment delivery. After completed treatment delivery, the same AP and lateral sequences were re‐acquired. Using orthogonal imaging, we evaluated the relationship between breathing signal and fiducial position in three orthogonal directions (LR, AP, and SI). Typically, data were acquired for 15–20 s per sequence. The goal was to acquire data for two to three full breathing cycles, but in some cases with irregular breathing, this was not feasible. Only sequences containing at least one full breathing cycle were used in the analysis. Fluoroscopic images were captured at 14.8 frames per second with a detector element size of 0.388 mm x 0.388 mm. The imaging source‐detector‐distance was 1500 mm and the treatment source‐axis‐distance distance 1000 mm.

The fluoroscopic images and the RPM data were exported to Matlab (version 2014b or higher, MathWorks, Natick, MA, USA) for analysis. Using an in‐house template‐matching algorithm based on the normalized cross‐correlation,^11^ we determined the center pixel position of each fiducial on every image. To make sure that fiducial positions were accurately tracked, we visually inspected all fiducial positions in all sequences.

### 2.5 Imaging geometry considerations

To accurately convert the projected fiducial locations on the x‐ray detector into positions in the patient coordinate system, we need to take the divergence of the x‐ray beam between the fiducials and the detector into account. As described in our previous study, we estimated one representative LR and one representative AP in‐room fiducial position using an iterative approach.^12^ We defined the LR axis as positive toward the left‐hand side of the patient, the AP axis as positive in the anterior direction, and the SI axis as positive in the superior direction.

### 2.6 Intrafractional relationship changes

**Figure 1:**
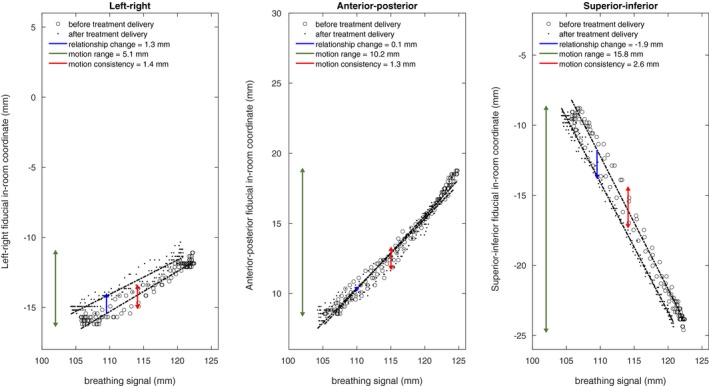
Illustration of the intrafractional relationship changes at 25% at the common breathing signal range for one fraction (blue arrows). Also shown are the fiducial motion ranges (green lines) and the fiducial motion consistency (red lines). Data are shown for one of the fiducials. Left‐hand side panel: left–right fiducial direction, Middle panel: anterior–posterior direction; Right‐hand side panel: superior–inferior direction.

We evaluated the IRC between the breathing signal and the fiducial positions in the AP, LR, and SI directions separately, and, whenever all four sets of images (AP and lateral before and after treatment delivery) had been acquired, the total 3D length of the changes (denoted 3D change). For each image sequence, we fit one linear curve between the RPM signal and SI fiducial positions, and one for either LR or AP fiducial positions (Fig. 1) depending on the imaging direction. To counteract the effect of irregular breathing cycles as well as uneven sampling of the breathing cycle, we made the linear fits equally weighted over the RPM range by stratifying the breathing signal into 1‐mm bins and fitting the curve to the average fiducial position within each bin. We calculated the IRC as the vertical distance between the two linear fits at 25% of the common breathing range (Fig. 1). We chose 25% because, in the case of 30–70% gating, it corresponds to fiducial positions close to the center of the gating window. Fig. 1 illustrates the quantification of the IRC between the breathing signal and the fiducial positions.

In some cases, the couch was used to reposition the patient between image acquisitions. For such fractions, we corrected for this before proceeding with the analysis. In cases with AP couch shifts, the RPM signal was changed accordingly.

The calculated relationship changes can be considered overall RPM‐fiducial relationship changes in that they will quantify all changes that occur during treatment delivery, including those introduced by patient shifts. To investigate intrafractional RPM‐fiducial relation changes excluding patient shifts, we estimated the shifts from the acquired image by calculating the average of the fluoroscopic images (typically > 200 images per acquisition) before and after treatment delivery. Image registration of the average images was performed on a central area containing one or more vertebrae. Then, we calculated the IRC as if the patient’s position had not changed during the treatment delivery. In cases with AP patient shifts, both the fiducial locations and the breathing signal were changed accordingly.^13^

### 2.7 Dose distribution impact

To estimate the dose distribution impact in the ITV, the PTV, and the OARs, we moved the patient isocenter according to the relationship changes and recalculated the dose distribution. We evaluated changes to the treatment plan objectives, and for the ITV, we evaluated changes to V_100%_.

### 2.8 Prediction of intrafractional relationship changes

To investigate whether it is possible to predict if an IRC occurs during treatment delivery without using additional imaging, we evaluated the absolute value of the baseline drift (BLD) of the breathing amplitude,^14^ the treatment delivery duration, the pretreatment fiducial motion ranges, and the fiducial motion consistency as potential predictors (see Fig. 1). The BLD was defined as the difference between the RPM signal at maximum exhale before and after treatment delivery. A negative BLD means a more posterior RPM box position after treatment delivery. We calculated the fiducial motion consistency as two standard deviations (SD) for fiducial positions in a 2‐mm window around the mid‐range RPM position.

We modeled the IRC in each direction for the four potential predictors using univariable linear regression. For fractions with a complete set of four image acquisitions, we also modeled the 3D IRC. In those cases, predictor values for both the motion range and the fiducial motion consistency were the corresponding 3D lengths. We also considered the squared Pearson correlation coefficient (R^2^) in our analysis.^11^ A *P*‐value below 0.05 was considered statistically significant.

## 3 RESULTS

### 3.1 Collected data

An overview of the fluoroscopic imaging and breathing signal sequences used in the analysis is shown in Table 1. A total of 38,147 images from 152 sequences were analyzed. For 31 fractions, data from all four image acquisitions (AP and lateral before and after treatment delivery) were available and could be used to calculate the 3D IRC and its estimated dosimetric impact.

**Table 1:** Fluoroscopic imaging and breathing signal data used in the analysis are denoted by X.

	Fraction 1				Fraction 2				Fraction 3				Fraction 4				Fraction 5			
	Before RT		After RT		Before RT		After RT		Before RT		After RT		Before RT		After RT		Before RT		After RT	
Patient id	AP	LAT	AP	LAT	AP	LAT	AP	LAT	AP	LAT	AP	LAT	AP	LAT	AP	LAT	AP	LAT	AP	LAT
1	X	X	X	X	X	X	X	X	X	X	X	X	X	X	X	X	X	X	X	X
2	X	X	X	X	X	X	X	X	‐	‐	‐	‐	‐	‐	‐	‐	‐	‐	‐	‐
3	X	X	X	X	‐	‐	‐	‐	X	X	X	‐	X	‐	X	X	X	‐	X	X
4	X	X	X	X	X	X	X	X	X	X	X	X	X	X	X	X	X	X	X	X
5	X	X	X	X	X	X	X	X	X	X	‐	X	X	X	X	X	X	X	X	X
6	X	X	X	X	X	X	X	X	X	X	X	X	X	X	X	X	X	X	‐	‐
7	X	X	‐	‐	X	X	X	X	X	X	X	X	X	X	X	X	X	X	X	X
8	X	X	X	X	X	‐	X	‐	X	‐	X	‐	‐	‐	‐	‐	X	‐	X	‐
9	X	X	X	X	X	X	X	X	X	X	X	X	‐	X	X	X	‐	X	X	X
10	‐	‐	‐	‐	X	X	X	X	‐	‐	‐	‐	‐	‐	‐	‐	X	X	X	X

RT, radiotherapy; AP, anterior–posterior imaging direction; LAT, lateral imaging direction.

### 3.2 Intrafractional relationship changes

**Figure 2:**
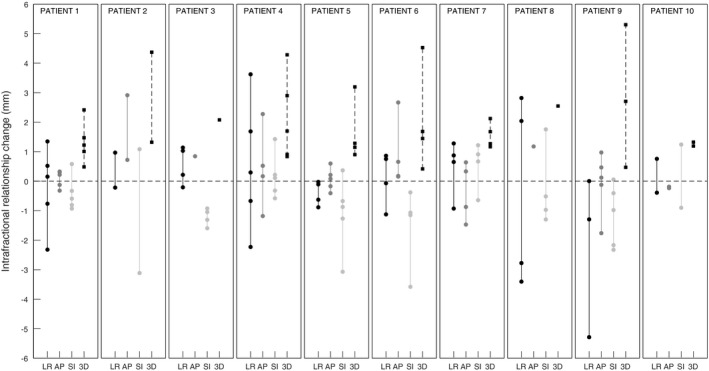
Intrafractional relationship change between the breathing signal and fiducial in‐room coordinates (average value for all implanted fiducials). For patients with more than one fiducial marker, the standard deviation of the relationship change for all the fiducials is typically less than 0.5 mm. LR, left–right; AP, anterior–posterior; SI, superior–inferior.

All calculated IRCs are shown in Fig. 2. The average (±1 SD) absolute intrafractional changes in the LR, AP, and SI directions were 1.2 ± 1.2 mm, 0.7 ± 0.7 mm, and 1.1 ± 0.8 mm, respectively. The average 3D relationship change was 2.0 ± 1.3 mm ranging from 0.4 to 5.3 mm. In five of 31 fractions, the 3D change was larger than 3 mm. These five occurrences took place in five separate patients (patient 2, 4, 5, 6, and 9 in Fig. 2). The median treatment delivery time was 8.5 min (range: 5.7–19.9 min).

For 70 pairs of LAT and AP sequences acquired immediately after one another (Table 1) with a median time between imaging of 0.9 min (range: 0.6–2.7 min), the average absolute SI relationship change was 0.6 ± 0.6 mm. The average absolute patient shifts in the LR, AP, and SI directions were 0.5 ± 0.7 mm, 0.4 ± 0.5 mm, and 0.3 ± 0.3 mm, respectively.

### 3.3 Dose distribution impact

**Figure 3:**
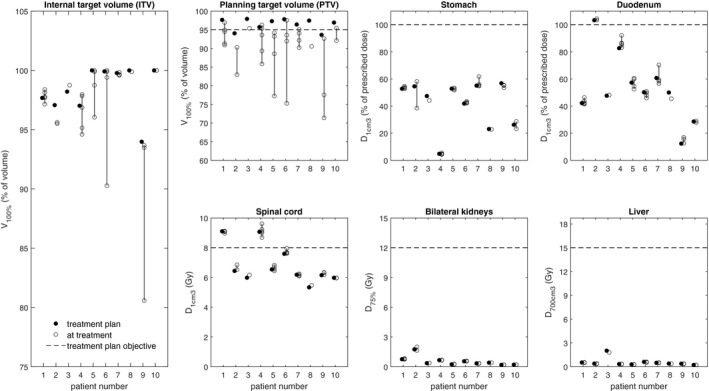
Dose distribution impact of the intrafractional relationship changes. The dashed lines show treatment plan objectives.

The dose distribution impact in target volumes and OARs is shown in Fig. 3. The IRC introduced degradation of ITV and PTV coverage. For the ITV V_100%_, the average (±1 SD) change was −1.1% ± 3.0% points with a range from −13.4 to +0.9. In three of 31 fractions, the ITV V_100%_ decreased by more than 3% points. This occurred when the IRC was larger than 4.3 mm and in three different patients. The average change for the stomach D_1cm3_ was −0.3% ± 3.5% points with a range from −16.1 to +6.7. The average change for the duodenum D_1cm3_ was 0.7% ± 3.5% points with a range from −4.8 to +9.6. For the spinal cord, the bilateral kidneys and the liver, all changes to the treatment plan objectives were within ±0.5 Gy.

### 3.4 Prediction of intrafractional relationship changes

**Figure 4:**
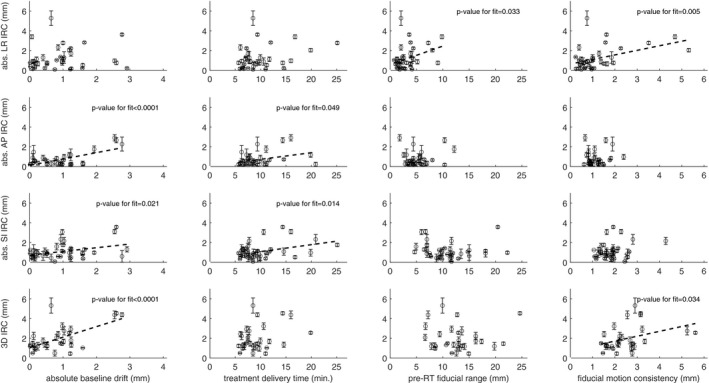
First row: Left–right (LR) intrafractional relationship changes (IRC) versus the four investigated predictors. Second row: Anterior–posterior (AP) IRC versus the four investigated predictors. Third row: Superior–inferior (SI) IRC versus the four investigated predictors. Fourth row: Total 3D IRC vs. the four investigated predictors. Linear fits for predictors statistically significant in univariable analysis are shown as dashed lines.

For the LR relationship change (Fig. 4, first row), the pre‐RT range and the fiducial motion consistency were statistically significant predictors in univariable analysis with *P*‐values of 0.033 and 0.005, respectively. The corresponding R^2^‐values were 0.12 for the range and 0.20 for motion consistency.

For the AP relationship change (Fig. 4, second row), the absolute BLD and the treatment delivery time were statistically significant predictors in univariable analysis with *P* < 0.001 and *P* = 0.049, respectively. The corresponding R^2^‐values were 0.42 for the BLD and 0.12 for treatment delivery time.

For the SI relationship change (Fig. 4, third row), the absolute value of the BLD and the treatment delivery time were statistically significant predictors in univariable analysis with *P*‐values of 0.021 and 0.014, respectively. The corresponding R^2^‐values were 0.13 for BLD and 0.15 for treatment delivery time.

For the 3D relationship change (Fig. 4, fourth row), the absolute BLD and the fiducial motion consistency were statistically significant predictors in univariable analysis with *P* < 0.001 and *P* = 0.034, respectively. The corresponding R^2^‐values were 0.39 for the BLD and 0.15 for the motion consistency.

**Figure 5:**
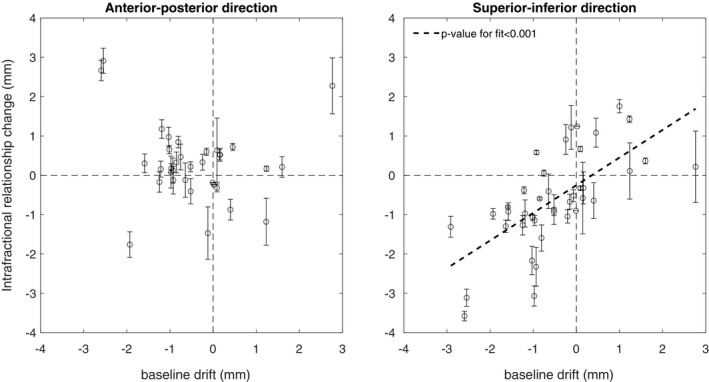
Intrafractional relationship changes versus the baseline drift. Left‐hand side: anterior–posterior direction. Right‐hand side: superior–inferior direction.

Given the impact of the absolute BLD on the absolute relationship change, we also analyzed the directional intrafractional change versus the BLD (Fig. 5). The relationship between the BLD and the SI shift was statistically significant (*P* < 0.001), whereas there was no statistically significant linear relationship in the AP direction.

## 4 DISCUSSION

In this study, we investigated the intrafractional consistency of the relationship between a breathing signal measured on the patient surface and the motion of fiducial markers in pancreatic cancer patients. Patients were treated with respiratory‐gated RT on a standard linac with a median treatment delivery time of 8.5 min. We quantified the relationship changes in the LR, AP, and SI directions separately and found that the IRC was somewhat larger in the LR and SI directions (1.1–1.2 mm) compared to the AP direction (0.7 mm). The average 3D IRC was 2.0 ± 1.3 mm, and in five of 31 cases larger than the 3‐mm ITV‐to‐PTV margin.

It should be noted that patients may move on the couch during treating delivery. We estimated patient movement by comparing the position of the vertebral column on the fluoroscopic images acquired before and after treatment delivery. The average measured absolute patient position shift was 0.3–0.5 mm in each direction. Because the breathing signal is measured as the AP amplitude of the patient posterior surface, an AP patient position shift may also offset the breathing signal, and, therefore, introduce IRC in all three directions (see Fig. 1). The effect is larger in the SI direction, where the slopes of RPM‐fiducial curves are the steepest (average slope: −1.2 mm/mm), and smaller in the LR direction, where the slopes typically are small (average slope: +0.1 mm/mm). In the AP direction, the influence will be limited, since the breathing signal and the fiducial shifts are observed in the same direction, partly canceling the effect. In this study, and since we aimed to quantify changes to the relationship between the breathing signal and the tumor position independently from patients’ movement, we compensated for changes in patient position in the analysis.^10^, ^15^

In three fractions with large IRC, the ITV coverage decreased by more than 3% points compared to the treatment plan. Since the dose gradients outside the PTV are typically steep in SBRT, IRCs larger than the ITV‐to‐PTV‐margin may result in compromised overall ITV coverage since lower doses from fractions with compromised coverage will not be compensated by larger doses from other fractions. The estimated dose distribution impact in the OARs was modest in most investigated cases, but a few occasions of larger impact was observed for the stomach and the duodenum due to their close proximity to the target volume. Nonsystematic shifts are equally likely in all directions, leading to higher doses from some fractions compensating lower doses from other fractions, and, therefore, resulting in little overall effect in patients treated with regularly fractionated RT. Patients treated with SBRT, however, are not necessarily subject to the same averaging properties as patients treated with regularly fractionated RT, and, especially given the large OAR doses per fraction, it is crucial to guarantee sufficient dose delivery accuracy at each fraction.

In the prediction analysis, IRC was found to be correlated with different metrics for different directions, indicating that the relationship between the breathing signal and the fiducial positions may be lost for various reasons. However, the metric most strongly associated with the 3D IRC was the baseline drift (BLD) of the breathing signal. For each millimeter of absolute BLD, the 3D IRC was about 1 mm (Fig. 4, the leftmost panel on the fourth row), which alone explained 39% of the IRC variance.

The relationships between the BLD and the relationship changes (Fig. 5) can be understood from the slopes of the RPM‐fiducial positions curves (Fig. 1). If the BLD is interpreted as a systematic offset of the RPM signal (not caused by a patient position shift) that occurred between pre‐ and post‐RT imaging, the BLD will horizontally shift the post‐RT linear fit, and the IRC (in each direction) will be ‐BLD × slope. This is closely related to the argument above regarding patient shifts in the AP direction.

The 2.0 ± 1.3 mm IRC for a median treatment time of 8.6 min from this study can be compared to the results reported by Malinovsky *et al.* 16 In their study, they reported that the prediction errors for the 3D fiducial position increased during the first 40 min of treatment delivery on a CyberKnife Synchrony. Their relationship between external and internal fiducial markers in the tumor was built during the first 10 min and had an average 3D prediction error of 1.2 mm. Applying this model during the subsequent 10–20‐, 20–30‐, and 30–40‐min blocks increased the average 3D errors to 2.0, 2.8 and 3.6 mm, respectively. In our study, we did not find a statistically significant relationship between treatment delivery time and 3D IRC (*P* = 0.074). There were, however, statistically significant relationships for the treatment delivery time for the AP IRC and the SI IRC (Fig. 4). It should be kept in mind that we investigated the IRC after removing the effect of patient shifts. The likelihood of uncorrected patient shifts occurring during treatment delivery increases with time,^9^ and such shifts will decrease treatment accuracy independently of IRC.

The risk that the relationship between an external breathing signal and the tumor location may change during treatment must be managed to ensure accurate dose delivery in all fractions. The only approach to finding out if changes have occurred is to periodically acquire new images of the patient in order to verify that the tumor is in its expected position. How often this should be done depends on the accuracy requirements of the treatment. Patients in our study were typically treated with hypofractionated SBRT to 9 Gy per fraction delivered by two 180‐degree VMAT arcs. If, for example, the relationship was checked by intrafractional imaging every 15 degrees, then, on average, 0.375 Gy would be delivered between image acquisitions. If such imaging is performed with kV images acquired perpendicular to the MV treatment beam (as it is on a Varian TrueBeam linac), the SI position of the fiducial markers could be found at any imaging angle. However, the LR/AP position cannot be found at arbitrary imaging angles without making further assumptions and/or using more advanced methods.^17^, ^18^

## 5 CONCLUSION

In this study of patients with pancreatic tumors treated with respiratory‐guided SBRT on standard linacs, we found that the relationship between an external breathing signal and the internal positions of fiducial markers changed 2 mm on average for a median treatment delivery time of 8.5 min. Part of this change could be explained by changes in the minimum breathing amplitude and the treatment delivery time. To safely deliver hypofractionated conformal treatments with small ITV‐to‐PTV margins, intrafractional imaging of the moving tumor is necessary to confirm that treatment accuracy is maintained.

## CONFLICTS OF INTEREST

Varian Medical Systems funded this study.
